# Supplementary material for: Asymmetric Synthesis of Quaternary Hydantoins via a Palladium-Catalyzed Aza-Heck Cyclization
Source: J Am Chem Soc. 2025 Nov 14;147(49):44692–8. doi: 10.1021/jacs.5c16022 (PMC12703750; doi:10.1021/jacs.5c16022)
Supplement: Supplementary file 2 [file ja5c16022_si_002.zip › All NMR FID Files/8/8_FluoroNMR/pdata/1/email_TDI01-064 xterize fluorine_3_1.pdf]

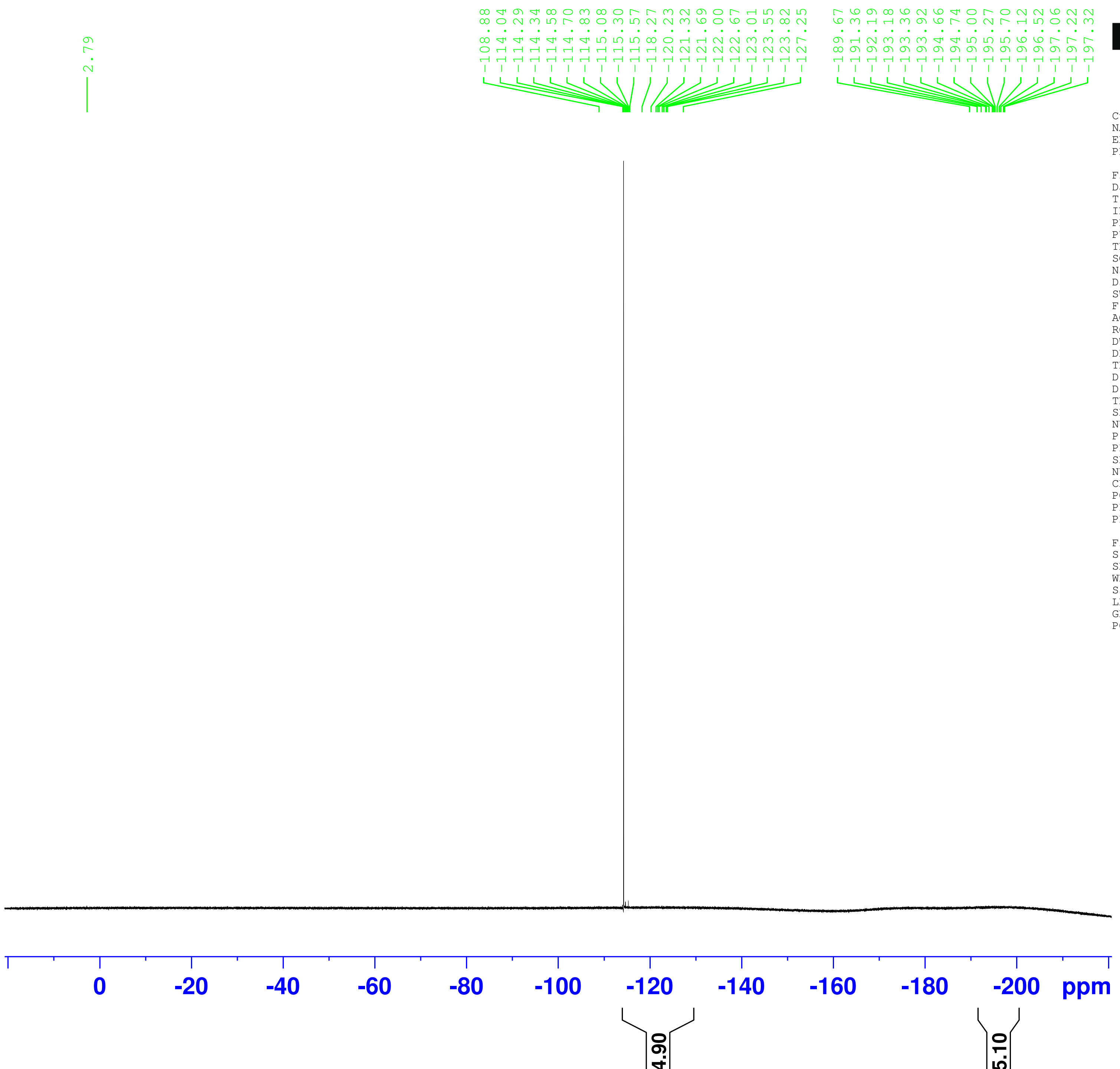

Current Data Parameters
NAME TDI01-064 xterize fluorine
EXPNO 3
PROCNO 1

F2 - Acquisition Parameters
Date_ 20250216
Time 4.46 h
INSTRUM Avance Neo 400 U of DE Brown Lab
PROBHD Z104450_0412 (
PULPROG zgig
TD 131072
SOLVENT CDCl3
NS 16
DS 4
SWH 90909.091 Hz
FIDRES 1.387163 Hz
AQ 0.7208960 sec
RG 101
DW 5.500 usec
DE 6.50 usec
TE 298.1 K
D1 1.00000000 sec
D11 0.03000000 sec
TD0 1
SFO1 376.4607164 MHz
NUC1 19F
P1 15.00 usec
PLW1 11.81599998 W
SFO2 400.1316005 MHz
NUC2 1H
CPDPRG[2 waltz16
PCPD2 90.00 usec
PLW2 8.31639957 W
PLW12 0.23101000 W

F2 - Processing parameters
SI 65536
SF 376.4983662 MHz
WDW EM
SSB 0
LB 0.30 Hz
GB 0
PC 1.00
